# Distinct seasonal migration patterns of Japanese native and non‐native genotypes of common carp estimated by environmental DNA

**DOI:** 10.1002/ece3.3346

**Published:** 2017-09-12

**Authors:** Kimiko Uchii, Hideyuki Doi, Hiroki Yamanaka, Toshifumi Minamoto

**Affiliations:** ^1^ Faculty of Pharmacy Osaka Ohtani University Tondabayashi Japan; ^2^ Graduate School of Simulation Studies University of Hyogo Kobe Japan; ^3^ Faculty of Science and Technology Ryukoku University Otsu Japan; ^4^ Graduate School of Human Development and Environment Kobe University Kobe Japan

**Keywords:** animal migration, environmental DNA, genotype, single nucleotide polymorphism

## Abstract

Understanding behavioral differences between intraspecific genotypes of aquatic animals is challenging because we cannot directly observe the animals underwater or visually distinguish morphologically similar counterparts. Here, we tested a new monitoring tool that uses environmental DNA (eDNA), an assemblage of DNA in environmental water, to specifically detect Japanese native and introduced non‐native genotypes of common carp (*Cyprinus carpio*) in Lake Biwa, Japan, and estimated differences between the two genotypes in the use of inland habitats. We monitored the ratios of native and non‐native single nucleotide polymorphism alleles of a mitochondrial locus of common carp in a lagoon connected to Lake Biwa for 3 years using eDNA. We observed seasonal dynamics in the allele frequency showing that the native genotype frequency peaked every spring, suggesting that native individuals migrated to the lagoon for spawning and then returned to the main lake, whereas non‐native individuals tended to stay in the lagoon. The estimated migration patterns corresponded with the estimates of a previous study, which were based on commercial fish catch data. Our findings suggest that eDNA‐based monitoring can be useful tool for addressing intraspecific behavioral differences underwater.

## 1 INTRODUCTION

Intraspecific behavioral differences driven by genetic variation are an important factor that affects ecological and evolutionary processes (Wolf & Weissing, 2012). However, in situ observation of such differences is generally very difficult in aquatic animals because of the inability to observe underwater animals and the difficulty in visually discriminating morphologically similar counterparts. A new development in monitoring animal distribution during the last decade can fill this methodological gap, as this method uses environmental DNA (eDNA) as a trace for animal presence.

eDNA is defined as DNA assemblages existing in environmental media, such as water and soil. As genetic assessment was first applied to eDNA recovered from water to estimate the distribution of bullfrogs (Ficetola, Miaud, Pompanon, & Taberlet, 2008), eDNA‐based monitoring of animal distribution has rapidly developed and gained general acceptance for use in detecting specific species (see reviews by Lodge et al., 2012; Rees, Maddison, Middleditch, Patmore, & Gough, 2014; Thomsen & Willerslev, 2015) and taxa (Miya et al., 2015; Thomsen et al., 2012; Valentini et al., 2016) in aquatic systems. The eDNA method can precisely identify species from genetic information, which is especially useful when dealing with similar‐looking organisms, such as intraspecific genotypes. We previously developed an eDNA method to quantify the relative biomass ratio of intraspecific genotypes of common carp (*Cyprinus carpio*) based on a single nucleotide polymorphism (SNP) (Uchii, Doi, & Minamoto, 2016). Here, we tested the potential use of this method to estimate the difference in the seasonal migration patterns of two genotypes of common carp.

As the Japanese archipelago broke apart from the Eurasian continent, a unique strain of common carp has evolved in Japan. Based on mitochondrial sequence data, the Japanese strain is estimated to have diverged from Eurasian ones ca. two million years ago (Mabuchi, Senou, Suzuki, & Nishida, 2005). However, domesticated Eurasian strains were introduced to Japan in 1905 at the latest (Maruyama, Fujii, Kijima, & Maeda, 1987), resulting in today's extensive invasion of non‐native common carp throughout Japanese freshwater systems (Mabuchi, Senou, & Nishida, 2008). Lake Biwa, the largest and oldest lake in Japan, is an important habitat for the Japanese native strain and other endemic freshwater fishes of Japan. An early report suggested that the native strain inhabited the main lake and migrated to littoral and inland habitats in the spring for spawning, whereas the non‐native strains inhabited the littoral and inland habitats all year round (Hurukawa, 1966). This estimate was based on only 1 year of commercial catch data in which the two strains were visually discriminated; thus, we still do not have concrete evidence about the migration patterns of native and non‐native strains.

In this study, we applied eDNA monitoring to estimate the seasonal use of inland habitats by the native and non‐native genotypes. The ratio of the two genotypes in a lagoon connected to Lake Biwa was monitored for 3 years using a previously developed eDNA assay that quantifies the ratio of native and non‐native DNA alleles based on a SNP (Uchii et al., 2016). We observed reproducible seasonal dynamics in the ratio of two genotypes, which suggested the differences in the migration patterns between the two genotypes.

## 2 MATERIALS AND METHODS

### 2.1 Water sampling and filtration for eDNA collection

**Figure 1:**
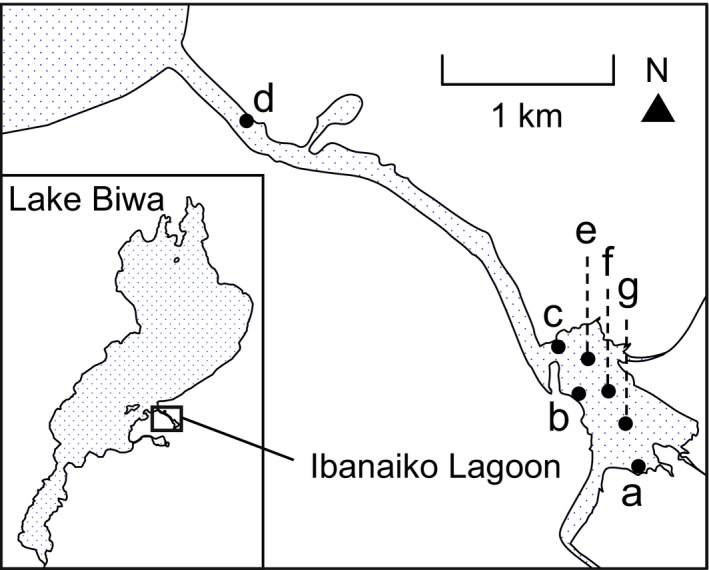
Sampling locations in Ibanaiko Lagoon, which is connected to Lake Biwa. Shore sites are labeled a–d and offshore sites are labeled e–g

Water sampling was performed in Ibanaiko Lagoon (Fig. 1), which is connected to Lake Biwa, Japan, 19 times between April 2013 and April 2016. To cover the wide range of the lagoon system, we set four monitoring sites on the shore, three of which were located from upstream to downstream in the lagoon (sites a–c; Fig. 1), and one of which was located in a channel flowing from the lagoon to the main lake (site d; Fig. 1). We collected 2 L of surface water at the four shore sites during every sampling occasion. In total, 76 samples (4 sites × 19 sampling times; Table 1) were collected on the shore. We also collected 2 L of surface water from three offshore sites (sites e–g; Fig. 1) during samplings between March 2015 and April 2016, except on 16 March 2016, to check the consistency of eDNA results between shore and offshore sites. In total, 21 offshore samples (3 sites × 7 sampling times; Table 1) were collected. Sampling bottles, which were carefully washed with detergent and distilled water in the laboratory, were rinsed with environmental water collected on site at least three times before sampling. Water samples were immediately placed in a cold box and transported to the laboratory within 5 hr. Each water sample was filtered onto a Whatman^®^ GF/F filter (47‐mm diameter, 0.7‐μm particle retention size; cat no. 1825‐04, GE Healthcare Japan, Tokyo, Japan) to capture eDNA. The filtered volume per filter ranged from 330 ml to 1 L but was 500 ml in most samples (see Table S1). The filtration apparatus was decontaminated with 5% bleach for at least 5 min and thoroughly washed with tap water and distilled water between samples. At the end of each sampling day, 1 L of distilled water was subjected to filtration in the same manner as the samples to control cross‐contamination during filtration and subsequent DNA extraction procedures. The filters were stored at −20°C until DNA extraction.

**Table 1:** Number of sampling times at shore (a–d in Fig. 1) and offshore (e–g in Fig. 1) sites each year

	2013	2014	2015	2016
Shore sites	6	5	4	4
Offshore sites	0	0	4	3

### 2.2 eDNA extraction from GF/F filters

DNA was extracted from each GF/F filter using the DNeasy^®^ Blood & Tissue Kit (Qiagen, Hilden, Germany). Each filter was soaked with 400 μl of Buffer AL and 40 μl of Proteinase K in an internal container of a Salivette^®^ tube (Cat no. SAR‐511534; Sarstedt, Nümbrecht, Germany) and incubated at 56°C for 30 min. After centrifugation at 5,000 *g* for 5 min, 220 μl of TE buffer (pH 8.0) was added to each filter in the tube, and the tubes were centrifuged again under the same conditions. Buffer AL (200 μl) and 100% EtOH (600 μl) were then added to each filtrate and mixed by pipetting. The mixture (~600 μl) was applied to a DNeasy Mini Spin Column and centrifuged at 6,000 *g* for 1 min. This step was repeated until the mixture was completely processed. We followed the manufacturer's instructions for further steps. Finally, DNA was eluted from the column with 100 μl of Buffer AE, with some exceptions (150 μl; Table S1).

### 2.3 Quantification of ratios of native and non‐native alleles in eDNA

Cycling probe technology is a highly sensitive method that uses DNA–RNA–DNA chimeric probes to detect SNPs; it involves using ribonuclease H to cut the RNA portion of the chimeric probe that hybridizes to the target DNA, thereby enabling sequence‐specific detection (Bekkaoui, Poisson, Crosby, Cloney, & Duck, 1996; Yatabe et al., 2006). We quantified the relative abundances of the DNA alleles of native and non‐native genotypes on the basis of SNP in the D‐loop region of mitochondrial DNA (mtDNA) using real‐time PCR with cycling probe technology according to Uchii et al. (2016). Briefly, we used two DNA–RNA–DNA probes labeled with different fluorescent dyes, one complementary to each of the genotypes, where the SNP base was composed of RNA as follows: native probe, 5′ Eclipse‐GAGATGCCAGA‐HEX 3′ (RNA, underlined); non‐native probe, 5′ Eclipse‐GAAATGCCAGA‐FAM 3′ (RNA, underlined). Using the StepOnePlus real‐time PCR system (Thermo Fisher Scientific, Waltham, MA, USA), we performed triplicate real‐time PCRs in a volume of 20 μl containing 1× Cycleave^®^PCR Reaction Mix (Takara Bio, Otsu, Japan), 200 nmol/L of the forward (5′‐TCCACCCTCGGATAAT‐3′) and reverse (5′‐ACTATGTAAGGATAAGTTGAACT‐3′) primers specific to *C. carpio*, 100 nmol/L of the native and non‐native probes, and 1–2 μl of template DNA under the following thermal cycling conditions: 30 s at 95°C, followed by 45 cycles of 5 s at 95°C, 10 s at 55°C, and 25 s at 72°C. The last base (A) at the 3′ end of the original reverse primer (Uchii et al., 2016) was removed to increase mismatches to related species within the last five bases at the 3′ end (Appendix  1). The standard mixtures of native and non‐native DNA (50–100 copies/reaction) in the ratios of 15:1, 7:1, 3:1, 1:1, 1:3, 1:7, and 1:15 (15, 7, 3, 1, 0.33, 0.14, and 0.07 in terms of native/non‐native DNA concentration ratios, respectively) were prepared, and at least five of them were included in triplicate or duplicate in every run (see details in Table S2). Using standard curves created between Δ*C*_T_ (calculated as *C*_T(non‐native DNA)_ – *C*_T(native DNA)_) and the native/non‐native DNA concentration ratios of the standard mixtures, the native/non‐native DNA concentration ratios of eDNA samples were quantified when the amplification signals were detected at *C*_T_ < 40 for both native and non‐native probes in at least two PCR replicates of a sample. A replicate in which either probe showed no signal was omitted from the calculation. At least two PCR blanks were included in every run to control for DNA contamination in the PCR. See Appendices 2 and 3 for the PCR inhibition test.

### 2.4 Quantification of common carp cytochrome *b* DNA in eDNA

When estimating the native and non‐native allele frequencies in the lagoon, we quantified the amount of common carp cytochrome *b* DNA (CytB), which was previously demonstrated to be linearly correlated with carp biomass in aquarium experiments (Takahara, Minamoto, Yamanaka, Doi, & Kawabata, 2012), to adjust for variations among sites in the amount of DNA. The copy numbers of CytB were quantified according to Takahara et al. (2012). Briefly, real‐time PCR was performed in triplicate in a reaction volume of 20 μl containing 1× TaqMan^®^ Fast Advanced Master Mix (Thermo Fisher Scientific), 900 nmol/L of the forward (CpCyB_496F: 5′‐GGTGGGTTCTCAGTAGACAATGC‐3′) and reverse (CpCyB_573R: 5′‐GGCGGCAATAACAAATGGTAGT‐3′) primers, 125 nmol/L of TaqMan probe (CpCyB_550P: 5′‐FAM‐CACTAACACGATTCTTCGCATTCCACTTCC‐TAMRA‐3′), and 1–2 μl of DNA template under the following thermal cycling conditions: 2 min at 50°C and 20 s at 95°C, followed by 45 cycles of 1 s at 95°C and 20 s at 60°C. The StepOnePlus real‐time PCR system was used for these procedures. A dilution series (3,000–1.5 copies/reaction) of linear plasmid containing the common carp CytB sequence was included in every run as a standard (see details in Table S3). The CytB concentrations of eDNA samples were quantified when the amplification signals were detected in at least two PCR replicates of a sample. The concentration of CytB at each site (copies/L) was calculated from the volumes of filtered water (330–1,000 ml) and eluted DNA (100–150 μl).

### 2.5 Estimation of native and non‐native genotype frequencies using eDNA

The ratio of native DNA was calculated for each eDNA sample as follows: ratio of native DNA = (native/non‐native DNA concentration ratio)/[1 + (native/non‐native DNA concentration ratio)]. Because the distribution of common carp eDNA can be heterogeneous even in a small lagoon (Takahara et al., 2012), we produced two different estimates for the frequency of native genotypes in Ibanaiko lagoon for each sampling day: (1) nonweighted frequency: we simply averaged the ratios of native DNA from the multiple sites on each sampling day, and (2) CytB‐weighted frequency: we calculated the weighted means of the ratios of native DNA from the multiple sites by incorporating the CytB concentration in each site to adjust for the differences in the amount of carp eDNA among the sites as ∑(ratio of native DNA × CytB concentration)_*i*_/∑(CytB concentration)_*i*_, where *i* indicates each sampling site on each sampling day. The CytB‐weighted frequency is theoretically equivalent to the frequency in an eDNA sample derived from a mixture of multiple water samples that are mixed at equal volumes. Note that the CytB adjustment was performed only within the same sampling date because seasonal differences in environmental factors affect eDNA persistence (e.g., water temperature affects eDNA degradation rates; Strickler, Fremier, & Goldberg, 2015). The time‐series data of the genotype frequencies for 3 years at each of the four shore sites were checked using a unit root test to discard the possibility of randomness. We performed the Phillips–Perron unit root test for the null hypothesis that the time series has a unit root against a stationary alternative. To check the possibility that the genotype frequencies were linearly decreased/increased over time, the genotype frequencies of the four shore sites were also analyzed by a binomial generalized linear model (GLM) with logit link function, with sampling year and site as explanatory factors. All statistical analyses were performed using R ver. 3.3.1 (R Core Team 2016),

## 3 RESULTS

### 3.1 Contamination during filtration, DNA extraction, and real‐time PCR

No amplification signals were detected in the eDNA extracts from the control GF/F filters used to filter 1 L of distilled water in the real‐time PCRs quantifying the native/non‐native DNA ratios and CytB. No amplification signals were detected in the PCR blanks in any real‐time PCR assays.

### 3.2 Dynamics of the frequencies of native and non‐native genotypes

**Figure 2:**
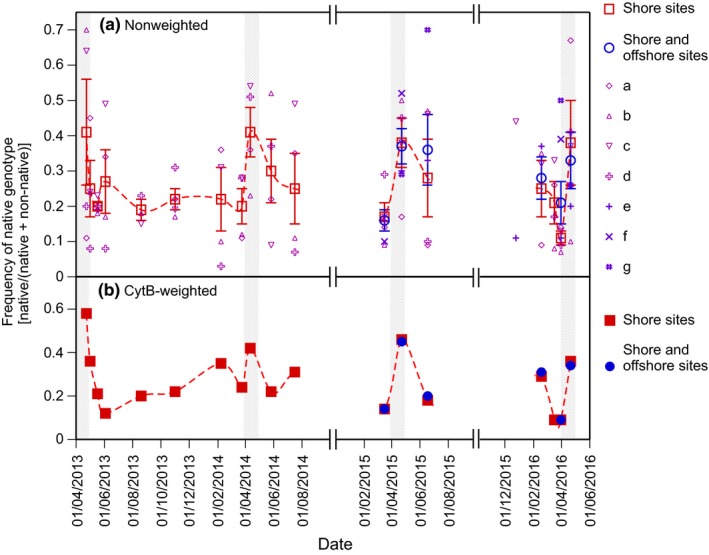
Dynamics of native genotype frequencies in Ibanaiko Lagoon. Shaded areas indicate breeding seasons. (a) Nonweighted frequencies of the native genotype in the lagoon estimated from the shore sites (open squares) and the shore and offshore sites combined (open circles). These frequencies were calculated when the ratios of native DNA were quantified at more than two sites on each sampling day. Vertical bars represent standard errors. The ratios of native DNA at each site (a–g) are also plotted. (b) CytB‐weighted frequencies of the native genotype in the lagoon estimated from the shore sites (solid squares) and the shore and offshore sites combined (solid circles). These frequencies were calculated when both the ratios of native DNA and the concentrations of CytB were quantified at more than two sites on each sampling day

The native/non‐native DNA concentration ratios, which were quantified based on the SNP in the D‐loop region, were successfully estimated for 85 of 97 samples (see Table S2 for *C*_T_ values and standard curves). The 12 eDNA samples that failed to meet estimates had low copy numbers of common carp mtDNA (i.e., <1 copy/μl of CytB). Figure 2 shows changes in the frequency of the native genotype in Ibanaiko lagoon over 3 years. The results of the Phillips–Perron unit root test on the time‐series data of native genotype frequencies at each of the four shore sites were significant (*p* < .05, Dickey–Fuller < −3.67 for all sites), indicating that the time‐series data of native genotype frequencies were not randomly determined. The GLM did not detect any significant effects of sampling year (Chi‐square = 0.19, *df* = 3, *p* > .9) or site (Chi‐square = 0.56, *df* = 3, *p* > .9) or interactions between these two factors (Chi‐square = 1.12, *df* = 9, *p* > .9), discarding the possibility that the genotype frequencies were linearly decreased/increased over time. The nonweighted frequencies of the native genotype estimated by a simple averaging of the ratios of native DNA at the shore sites showed repetitive seasonal dynamics in which the frequencies were highest in the spring when spawning occurred and lower in other seasons (Fig. 2a, plotted as open squares). The nonweighted frequencies of the native genotype estimated from the shore and offshore sites combined (Fig. 2a, open circles) showed similar values to those estimated from the shore sites alone (open squares). The CytB‐weighted frequencies of the native genotype (Fig. 2b) showed slightly different dynamics compared with the nonweighted frequencies. However, the overall trend was similar, as the CytB‐weighted frequencies of the native genotype were also highest in the spring every year. The CytB‐weighted frequencies estimated from the shore sites alone (Fig. 2b, solid squares) showed similar values to those estimated from the shore and offshore sites combined (solid circles).

## 4 DISCUSSION

We estimated the differences in the seasonal use of inland habitats between Japanese native and introduced non‐native genotypes of common carp in Lake Biwa by monitoring SNP allele frequencies in eDNA. Samples collected over 3 years, including four springs, showed repetitive seasonal dynamics in the frequency of the native genotype for both the nonweighted (Fig. 2a, open squares) and CytB‐weighted (Fig. 2b, solid squares) estimates. The native genotype frequencies peaked every spring in the lagoon (Fig. 2), suggesting individuals with the native genotype likely to migrate to the lagoon for spawning. The native genotype frequency decreased after the spawning season, suggesting that many individuals with the native genotype left the lagoon and moved, presumably to the main lake, whereas non‐native individuals tended to remain in the lagoon. Such a difference in migration patterns between the native and non‐native genotypes corresponds well to the previous estimate based on commercial fish catch data (Hurukawa, 1966). The latter showed that the catch amount of the native strain increased in the spring and decreased after the summer in littoral and inland habitats and increased in the winter in pelagic habitats. However, the catch amount of the non‐native strains did not fluctuate substantially in the littoral and inland habitats and was very low in the pelagic habitats throughout the year (Hurukawa, 1966). Furthermore, the seasonal changes in the native genotype frequency observed in the present study were roughly consistent with the changes detected in common carp individuals that were caught in Ibanaiko lagoon in a previous study (Uchii, Okuda, Minamoto, & Kawabata, 2013; see Appendix  4). These observations suggest the potential use of eDNA for detecting behavioral differences between closely related species.

When we deal with morphologically similar organisms, such as intraspecific genotypes and subspecies, there is often difficulty in visual discrimination. The eDNA method can readily discriminate genotypes because it uses genetic information. Furthermore, we can obtain long‐term data of genotype frequencies with substantially less effort than is needed for traditional catch sampling or biotelemetry (i.e., genetic analyses of large numbers of individual fish after catch or long‐term tracking of large numbers of individuals after tagging them, respectively). As research generally requires long‐term observations to detect seasonal patterns of animal migration, the time‐ and cost‐effectiveness of the eDNA method would be a great advantage.

On small spatial or short temporal scales, there is difficulty in linking eDNA information with animal presence and biomass due to eDNA dispersal, eDNA degradation, and animal movement (Barnes & Turner, 2016; Barnes et al., 2014). Thus, eDNA monitoring would be more suitable for tracking animal movement at larger spatial and temporal scales, such as seasonal migration, because the dispersal range of eDNA and the stability of DNA molecules are limited in the environment (e.g., Barnes et al., 2014; Jane et al., 2015; Maruyama, Nakamura, Yamanaka, Kondoh, & Minamoto, 2014; Strickler et al., 2015). A recent study demonstrated that the existence of target DNA was correlated with the migration range of anadromous fish (Yamanaka & Minamoto, 2016). Another study demonstrated a significant positive correlation between DNA concentration and the number of bighead carp detected by telemetry in a spawning habitat in which the increase in DNA was presumably attributable to the individuals that migrated for spawning (Erickson et al., 2016). These studies, together with the present study, suggest the great potential of the eDNA method for monitoring seasonal animal movement.

Most eDNA studies conducted thus far have used mtDNA markers to increase the detection probability because the copy number of mtDNA is much greater than that of nuclear DNA. We also targeted mtDNA for the same reason. However, mtDNA markers have a disadvantage in that they do not distinguish hybrids due to the maternal inheritance of mitochondria. As intraspecific genotypes can hybridize, we cannot exclude the possibility that the native genotype frequency based on mtDNA haplotypes would be over‐ or underestimated if cytonuclear disequilibrium was to exist. The use of nuclear DNA markers would solve this problem. Although several nuclear DNA markers that distinguish the Japanese native and non‐native genotypes of common carp have been reported (Mabuchi, Song, Takeshima, & Nishida, 2012), these markers are single‐copy and thus very difficult to detect in eDNA. However, because these markers can detect intraspecific genetic structure, the development of an effective eDNA concentration method to detect single‐copy genes would greatly expand the scope of eDNA studies. Alternatively, the development of markers in multiple‐copy nuclear genes would offer great potential for evaluating intraspecific genetic structure. Nuclear ribosomal DNA (rDNA) would be a strong candidate, as a recent study demonstrated that a marker in the internal transcribed spacer region of rDNA has greater sensitivity than a mtDNA marker in eDNA detection (Minamoto et al., 2017). As many eDNA studies have repeatedly noted, the greatest advantage of eDNA monitoring is the simplicity of its sampling and analysis procedures, which substantially saves time, labor, and costs and enables long‐term and/or wide‐range surveys. Given that eDNA monitoring requires only water sampling in the field, it can be applied to species that are endangered or difficult to catch, on which conventional catch sampling and biotelemetry are not easy to perform. Thus far, eDNA monitoring has developed mainly in the field of conservation. Future development of eDNA monitoring may also be directed toward application of this technique to the detection of ecological events, such as animal migration. eDNA monitoring, together with other methods, such as biotelemetry and catch sampling, will enhance our understanding of underwater animal behavior.

## CONFLICT OF INTEREST

None declared.

## AUTHOR CONTRIBUTIONS

K.U., H.D., H.Y., and T.M. conceived and designed the study. K.U. and H.Y. performed the experiments, and K.U. and H.D. analyzed the data. K.U., H.D., H.Y., and T.M. wrote the manuscript.

## DATA ACCESSIBILITY

Threshold cycle data of real‐time PCR assays: Tables S2 and S3.

## Figures and Tables

**APPENDIX 1:**
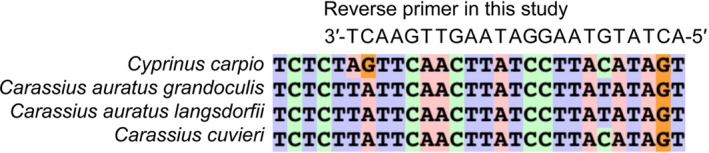
Reverse primer to amplify the SNP locus in the D‐loop region of mtDNA used in this study. The sequence was modified from the original reverse primer in Uchii et al. (2016).

**APPENDIX 2:**
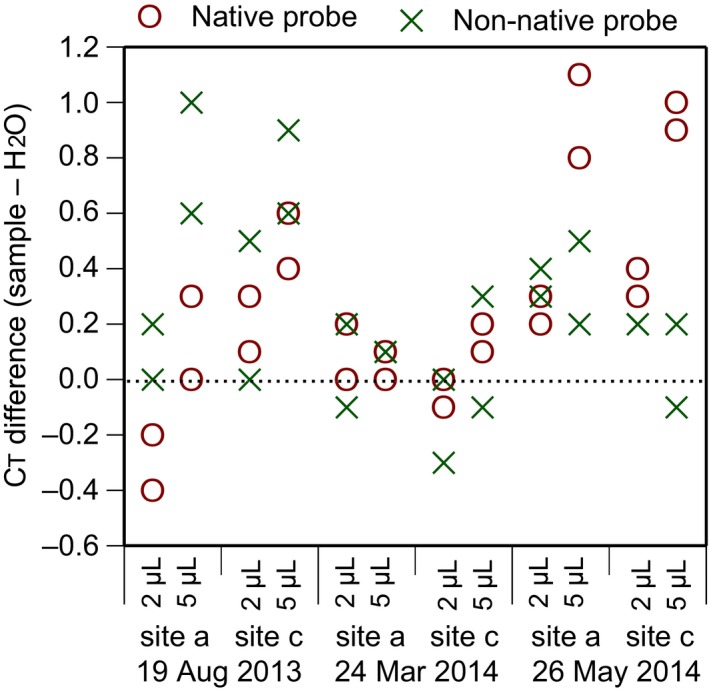
Shift of threshold cycle (*C*_T_) values in field eDNA samples spiked with the standard DNA mixture of native and non‐native DNA at a ratio of 1:1 (100 copies each/reaction). The copy number of the spiked DNA was ca. >10 times larger than that of target the DNA originally contained in the filed samples. Real‐time PCR was performed in duplicate. Delayed *C*_T_ values were observed in some samples when 5 μl of eDNA was used as template, whereas little or no inhibition effect was detected with 2 μl of template.

**APPENDIX 3:**
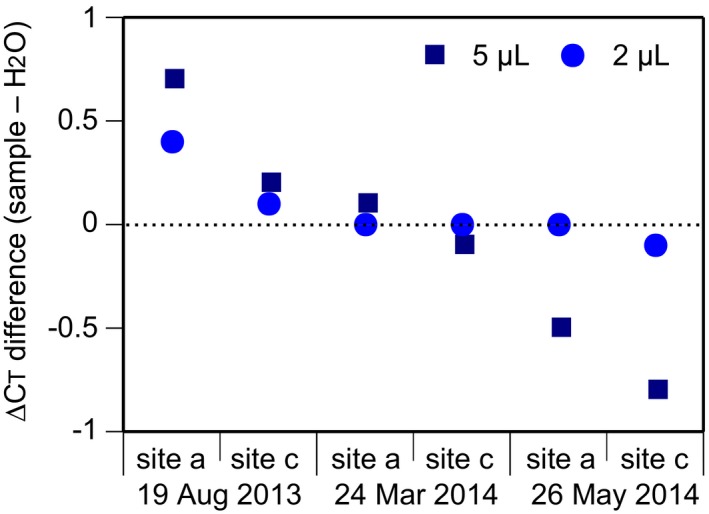
Shift of Δ*C*_T_ (*C*_T(non‐native)_ – *C*_T(native)_) in field eDNA samples spiked with the standard DNA mixture, as in Appendix  2. Duplicate *C*_T_ values of each sample were averaged before the Δ*C*_T_ calculation. Shift of Δ*C*_T_ values was detected in some samples when 5 μl of eDNA was used as template, whereas no or little shift was detected when 2 μl of eDNA was used. Together with the results in Appendix 2, we decided to use ≤2 μl of eDNA as template in the real‐time PCR assays

**APPENDIX 4:**
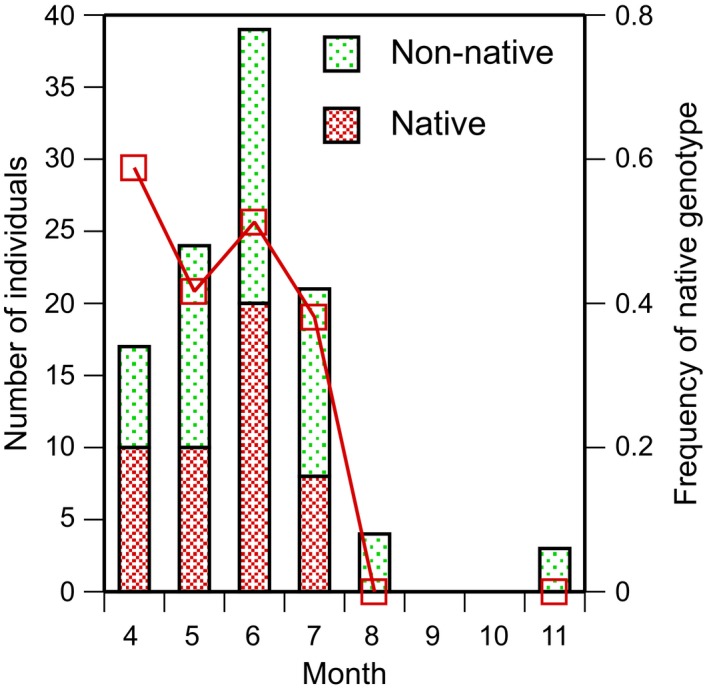
Frequency of the native genotype by month for common carp (≥350 mm in standard length and appeared to be adult) captured in Ibanaiko Lagoon in 2006, 2008, and 2009 in Uchii et al. (2013). Because common carp are no longer commercially fished in this area, all carp were caught as bycatch. Note that the native strain of common carp is more susceptible to the lethal pathogen *Cyprinid herpesvirus 3*, which was introduced to Lake Biwa in 2003, than are the non‐native strains (Ito, Kurita, & Yuasa, 2014); thus, the proportion of the native strain in Lake Biwa is likely to gradually decline with time (Uchii et al., 2013)
